# Occurrence of SARS-CoV-2 viremia is associated with genetic variants of genes related to COVID-19 pathogenesis

**DOI:** 10.3389/fmed.2023.1215246

**Published:** 2023-09-22

**Authors:** Emilia Roy-Vallejo, Sara Fernández De Córdoba-Oñate, Pablo Delgado-Wicke, Ana Triguero-Martínez, Nuria Montes, Rosa Carracedo-Rodríguez, Nelly Zurita-Cruz, Ana Marcos-Jiménez, Amalia Lamana, José María Galván-Román, Gonzalo Villapalos García, Pablo Zubiaur, Marianela Ciudad, Laura Rabes, Marta Sanz, Carlos Rodríguez, Almudena Villa, Jesús Álvarez Rodríguez, Celeste Marcos, Julia Hernando, Paula Díaz-Fernández, Francisco Abad, Ignacio de los Santos, Diego A. Rodríguez Serrano, Rosario García-Vicuña, Carmen Suárez Fernández, Rosa P. Gomariz, Cecilia Muñoz-Calleja, Elena Fernández-Ruiz, Isidoro González-Álvaro, Laura Cardeñoso, the PREDINMUN-COVID Group, Ana Barrios, Jesús Sanz, Pedro Casado, Ángela Gutiérrez, Azucena Bautista, Pilar Hernández, Nuria Ruiz Giménez, Berta Moyano, Paloma Gil, María Jesús Delgado, Pedro Parra, Beatriz Sánchez, Carmen Sáez, Marta Fernández Rico, Cristina Arévalo Román, Santos Castañeda, Irene Llorente, Eva G. Tomero, Noelia García Castañeda, Miren Uriarte, Leticia Fontán García-Rodrigo, Diego Domingo García, Teresa Alarcón Cavero, María Auxiliadora Semiglia Chong, Ainhoa Gutiérrez Cobos, Francisco Sánchez-Madrid, Enrique Martín Gayo, Ildefonso Sánchez-Cerrillo, Pedro Martínez-Fleta, Celia López-Sanz, Ligia Gabrie, Luciana del Campo Guerola, Reyes Tejedor, Julio Ancochea, Elena García Castillo, Elena Ávalos, Ana Sánchez-Azofra, Tamara Alonso, Carolina Cisneros, Claudia Valenzuela, Francisco Javier García Pérez, Rosa María Girón, Javier Aspa, Celeste Marcos, M. del Perpetuo Socorro Churruca, Enrique Zamora, Adrián Martínez, Mar Barrio Mayo, Rosalina Henares Espi, Rosa Méndez, David Arribas, Marta Chicot Llano, Begoña González, Begoña Quicios, Pablo Patiño, Marina Trigueros, Cristina Dominguez Peña, David Jiménez Jiménez, Pablo Villamayor, Alfonso Canabal, Rafael de la Cámara, Javier Ortiz, Isabel Iturrate

**Affiliations:** ^1^Internal Medicine Department, Hospital Universitario La Princesa, Madrid, Spain; ^2^Instituto de Investigación Sanitaria La Princesa (IIS-IP), Madrid, Spain; ^3^Rheumathology Department, Hospital Universitario La Princesa, Madrid, Spain; ^4^Molecular Biology Unit, Hospital Universitario La Princesa, Madrid, Spain; ^5^Microbiology Department, Hospital Universitario La Princesa, Madrid, Spain; ^6^Immunology Department, Hospital Universitario La Princesa, Madrid, Spain; ^7^Cell Biology Department, Facultad de Biología, Universidad Complutense, Madrid, Spain; ^8^Clinical Pharmacology Department, Hospital Universitario La Princesa, Instituto Teófilo Hernando, Universidad Autónoma de Madrid (UAM), Madrid, Spain; ^9^Pneumology Department, Hospital Universitario La Princesa, Madrid, Spain; ^10^Anesthesiology Department, Hospital Universitario La Princesa, Madrid, Spain; ^11^Centro de Investigación Biomédica en Red de Enfermedades Hepáticas y Digestivas (CIBERehd), Instituto de Salud Carlos III, Madrid, Spain; ^12^Centro de Investigación Biomédica en Red en Enfermedades Infecciosas (CIBERINFEC), Instituto de Salud Carlos III, Madrid, Spain; ^13^Intensive Care Unit, Hospital Universitario La Princesa, Madrid, Spain; ^14^Universidad Autónoma de Madrid (UAM), Madrid, Spain

**Keywords:** SARS-CoV-2, viremia, COVID-19, single nucleotide polymorphism (SNPs), genetic variants

## Abstract

**Introduction:**

SARS-CoV-2 viral load has been related to COVID-19 severity. The main aim of this study was to evaluate the relationship between SARS-CoV-2 viremia and SNPs in genes previously studied by our group as predictors of COVID-19 severity.

**Materials and methods:**

Retrospective observational study including 340 patients hospitalized for COVID-19 in the University Hospital La Princesa between March 2020 and December 2021, with at least one viremia determination. Positive viremia was considered when viral load was above the quantifiable threshold (20 copies/ml). A total of 38 SNPs were genotyped. To study their association with viremia a multivariate logistic regression was performed.

**Results:**

The mean age of the studied population was 64.5 years (SD 16.6), 60.9% patients were male and 79.4% white non-Hispanic. Only 126 patients (37.1%) had at least one positive viremia. After adjustment by confounders, the presence of the minor alleles of rs2071746 (*HMOX1*; T/T genotype OR 9.9 *p* < 0.0001), rs78958998 (probably associated with *SERPING1* expression; A/T genotype OR 2.3, *p* = 0.04 and T/T genotype OR 12.9, *p* < 0.0001), and rs713400 (eQTL for *TMPRSS2*; C/T + T/T genotype OR 1.86, *p* = 0.10) were associated with higher risk of viremia, whereas the minor alleles of rs11052877 (*CD69*; A/G genotype OR 0.5, p = 0.04 and G/G genotype OR 0.3, *p* = 0.01), rs2660 (*OAS1*; A/G genotype OR 0.6, *p* = 0.08), rs896 (*VIPR1*; T/T genotype OR 0.4, *p* = 0.02) and rs33980500 (*TRAF3IP2*; C/T + T/T genotype OR 0.3, *p* = 0.01) were associated with lower risk of viremia.

**Conclusion:**

Genetic variants in *HMOX1* (rs2071746), *SERPING1* (rs78958998), *TMPRSS2* (rs713400), *CD69* (rs11052877), *TRAF3IP2* (rs33980500), *OAS1* (rs2660) and *VIPR1* (rs896) could explain heterogeneity in SARS-CoV-2 viremia in our population.

## 1. Introduction

Almost three years after the SARS-CoV-2 pandemic outbreak, nearly 780 million people have been infected and 65% of the worldwide population has been fully vaccinated against COVID-19. Despite this fact, SARS-CoV-2 circulation seems to persist worldwide and still 1,500 people die every week due to COVID-19 (1). The wide spectrum of clinical manifestations of the disease has encouraged scientists to keep studying different biomarkers that could help us to achieve an early stratification of those patients at higher risk of respiratory impairment and death. In this regard, many clinical conditions and biomarkers such as age, hypertension, Interleukin-6 (IL-6) or D-dimer are associated with COVID-19 severity (2, 3) but the prediction of the clinical course and the pre-existing conditions that confer increased risk, remain a challenge for physicians.

In this sense, SARS-CoV-2 RNA detection in peripheral blood (viremia) has been proposed as a risk factor for severe COVID-19. In previous studies of our group, we have shown that patients with SARS-CoV-2 viremia were more likely to die or be admitted to the Intensive Care Unit (ICU) (4, 5). Other studies (6–8), including a meta-analysis (9) have confirmed these findings correlating SARS-CoV-2 viremia with worse COVID-19 prognosis. Viremia is associated with an increase in the inflammatory response, with higher levels of C-reactive protein or IL-6, as described by Hagman et al. (10) and Myhre et al. (11). In a proteomic study Li et al., evaluated pathways related to the development of viremia and found that patients with viremia had higher expression of SARS-CoV-2 entry factors (ACE2, CTSL, FURIN), proinflammatory markers (such as IL6) as well as markers of tissue damage and coagulation (12). Nevertheless, the mechanisms and predisposing factors, such as genetic factors, leading to viremia are not clear yet.

Several studies have assessed the association between genetic variants and COVID-19 prognosis by Single Nucleotide Polymorphism (SNP) genotyping and Genome-Wide Association Studies (GWAS). Two of the most studied genes are *ACE2* and *TMPRSS2*, involved in SARS-CoV-2 entry, and some of their genetic variants have been associated with COVID-19 severity and infectivity (13–16). Moreover, other regions of genetic susceptibility for COVID-19 severity have been described, such as those related to the ABO blood group system or the antiviral response (OAS1, OAS2, OAS3, TYK2, IFNAR2 or IL-10) (17–19). The review by Anastassopoulou et al. described how disease severity is determined by variants of genes involved in the immune response to the virus, while susceptibility to infection is mainly related to genes that participate in the early stages of infection (such as virus binding and entry) (20). Although these variants could potentially lead to increased entry and dissemination of the virus into the bloodstream, to date no study has addressed the relationship between genetic variants of genes involved in COVID-19 pathogenesis and the detection of viremia.

The main aim of this study was to evaluate the relationship between SARS-CoV-2 viremia and several SNPs in genes previously studied by our group as predictors of COVID-19 severity (13).

## 2. Materials and methods

### 2.1. Study design, population and data collection

This is a subanalysis of two previous studies assessing the relationship between different genetic variants related to the pathogenesis of SARS-CoV-2 and COVID-19 severity (13). Both were retrospective observational studies including patients attended at the University Hospital La Princesa (Madrid).

The first study (hereafter study A) recruited 817 patients from the first months of the pandemic (March 29th – April 29th 2020) and studied 120 SNPs that had previously been related to COVID-19, the coagulation cascade and the metabolism of COVID-19 treatments (Supplementary Table S1) (13). The second study (from now on study B) included 1,350 patients between March 29th 2020 and December 31st 2021 and mainly focused on 29 SNPs related to the regulation of immune, complement and coagulation pathways (Supplementary Table S2) (manuscript in preparation).

To enroll a significant number of patients with viremia determination that would enable assessment of the relationship between viremia and COVID-19 related SNPs all participants in studies A and B who had been checked for the presence of viremia, at least one time in the first week of hospitalization (*n* = 340) were selected for the current study (Supplementary Figure S1). All patients were older than 18 years and had confirmed SARS-CoV-2 infection (RT-PCR, antigen or serological testing).

Blood samples for genotyping were collected during hospitalization since all patients included in this study required admission. Plasma samples for viremia quantification were collected during the first week of admission, following the hospital protocols and the criteria of the physician in charge.

All data were collected from the clinical charts and included in an anonymized electronic database.

### 2.2. Selection of the SNPs genotyped

A total of 38 SNPs were genotyped in the whole population of the study (Supplementary Table S3). As the 340 patients tested for viremia were part of study B (manuscript in preparation), the 29 SNPs analyzed in that study were included in the current manuscript. In study A, 120 SNPs were analyzed (13). However only 107 of the 340 patients were included in study A. Since we could not genotype these 120 SNPs in the remaining 233 patients due to the high cost, we performed a pre-analysis of the importance of these 120 SNPs among the 107 patients included in study A, selecting those with *p* < 0.15, as described below in the statistical analysis section. We selected 9 SNPs which were later genotyped in the remaining 233 patients. Therefore, these 9 SNPs from study A were added to the 29 SNPs from study B.

### 2.3. Genotyping

In study A, a Maxwell RSC automated DNA extractor (Promega) was used to extract DNA from peripheral blood. A customized genotyping array was designed and the genotype analysis was performed with a QuantStudio 12 K flex thermal cycler along with an OpenArray thermal block (Thermo Fisher Scientific). In study B (ongoing manuscript), DNA was extracted using MagNA Pure 2.0 and MagNA Pure LC DNA Isolation Kit (Roche Life Science, Basel Switzerland). To genotype the selected SNPs, qPCR was performed using QuantStudio 12 k, TaqManTM Genotyping Master Mix and TaqManTM customized 384 plates (ThermoFisher Scientific, Waltham, MA) in Parque Científico of Universidad Autónoma de Madrid. Allelic discrimination was based on allele-specific fluorescence, which was automatically defined by TaqMan SNP Genotyping App (Applied Biosystems Software). To verify assay’s accuracy, negative controls and duplicate samples were used.

The candidate SNPs selected from the study A were genotyped by qPCR using a predesigned single nucleotide polymorphism (SNP) Genotyping Taqman Assays (Applied Biosystems, Waltham, MA. Part number in Supplementary Table S3). The assay was carried out following the manufacturer’s recommendations; duplicate samples and negative controls were also included to check the accuracy of the genotyping. Each sample’s genotype was determined automatically by measuring allele-specific fluorescence on a CFX Touch Real-Time PCR System using the software CFX 3.1 Manager (BioRad, Hercules, CA, United States).

### 2.4. SARS-CoV-2 RNA extraction, detection and quantification

SARS-CoV-2 viremia was detected by quantitative RT-PCR (QuantStudio™ 5 Real-Time PCR System) (Applied Biosystems) using the TaqPath™ COVID-19 CE IVD RT-PCR kit (Thermo Fisher Scientific). Amplification curves were analyzed with QuantStudio™ Design and Analysis software version 2.4.3 (Applied Biosystems). All plasma samples were included in duplicates in the assay. Viral load quantification was obtained by plotting Ct values through the standard curve and only viremias with mean Ct ≤37 (approximately 1.3 log 10, namely 20 copies/mL) and standard deviation (SD) <0.5 in the duplicate test for each gene were considered quantifiable.

### 2.5. Variables

The main outcome of this study was the detection of SARS-CoV-2 viremia in the first week of hospitalization. A positive viremia was defined as the presence of at least one determination with a viral load above the quantification threshold (20 copies/mL).

Age was considered as an ordinal qualitative variable and was categorized in three groups: <45 years, 45–70 years and > 70 years.

Severe COVID-19 was defined as the need for mechanical ventilation (invasive or non-invasive), high-flow oxygen, or death.

### 2.6. Statistical analysis

Quantitative variables were expressed as mean and standard deviation (SD) or median and interquartile range (IQR) for the variables with non-normal distribution. For qualitative variables, frequency and proportions were used. To analyze statistical differences between variables, Student’s t test, Mann–Whitney or Kruskall-Wallis tests were performed for quantitative variables, and χ2 test for qualitative variables.

The selection of the candidate SNPs from study A to genotype in the remaining 233 patients was performed by analyzing the most relevant SNPs in the 107 patients who had all the SNPs genotyped. To this end, the clinical variables associated with viremia in the bivariate analysis in these 107 patients were included in a multivariate logistic regression analysis. Then, each SNP was forced in the model. SNPs with a *p* < 0.15 in the model were selected. Also, an analysis of the variance of the model of each SNP was performed to help make the selection.

Finally, to determine which clinical variables were associated with the presence of viremia, a multivariate logistic regression analysis was performed (Supplementary Table S4). It was first modeled by adding all the variables with a *p* value lower than 0.15 in the bivariate analysis. The final clinical model was reached through backward stepwise removal of variables with p value higher than 0.15. Then, all SNPs were independently included in the clinical model. Those SNPs reaching a p value lower than 0.15 were included together in the final clinical model in order to analyze interactions between them. As previously described, we used a stepwise backwards approach to design the best model for predicting viremia. Then, the jackknife method was applied to reduce bias.

All the analyses were performed with Stata 14.0 for Windows (Stata Corp LP, College Station, TX, United States). Figures were depicted with R Studio (R Core Team 2022. R: A language and environment for statistical computing. R Foundation for Statistical Computing, Vienna, Austria).

### 2.7. Ethics

This study followed the ethical principles of the Declaration of Helsinki and it was approved by the Research Ethics Committee of University Hospital La Princesa, Madrid, (register numbers 4,111 and 4,070). All patients, except those who died, gave oral or written consent to participate, which was registered in their electronic clinical chart. Due to the COVID-19 pandemic emergency, oral consent was accepted as proposed by the AEMPS (Agencia Española de Medicamentos y Productos Sanitarios, The Spanish Agency for Medicines and Medical Devices).

## 3. Results

### 3.1. Clinical variables associated with SARS-CoV-2 viremia

The study population included 60.9% male and 79.4% white non-Hispanic patients, with a mean age of 64.5 years (SD 16.6). The most frequent comorbidities were hypertension (40.3%), dyslipidemia (38.5%), obesity (15.9%) and diabetes mellitus (15.4%) as shown in Table 1. Treatment during hospitalization and analytical variables are shown in Supplementary Table S5.

**Table 1:** Demographic and clinical data by viremia status.

	Study population (*n* = 340)	No viremia (*n* = 214)	Viremia (*n* = 126)	*p* value
Age; mean (SD)	64.5 (16.6)	63.5 (18.3)	66 (13.2)	0.19
Male sex; *n* (%)	207 (60.9)	116 (54.2)	91 (72.2)	0.001
Race/ethnicity; *n* (%)				
White, non-Hispanic	270 (79.4)	164 (76.6)	106 (84.1)	0.07
White, Hispanic	63 (18.5)	47 (22)	16 (12.7)	
Afrodescendent	1 (0.3)	1 (0.5)	0	
Asian	6 (1.8)	2 (0.9)	4 (3.2)	
Hypertension; *n* (%)	137 (40.3)	81 (37.9)	56 (44.4)	0.23
Dyslipidemia; *n* (%)	131 (38.5)	74 (34.6)	57 (45.2)	0.05
Diabetes mellitus				
Without organ damage	41 (12.2)	22 (10.3)	19 (15.1)	0.43
With organ damage	11 (3.2)	7 (3.3)	4 (3.2)	
Obesity; *n* (%)	54 (15.9)	34 (15.9)	20 (15.9)	1
Dementia; *n* (%)	14 (4.1)	12 (5.6)	2 (1.6)	0.07
Chronic Obstructive Pulmonary Disease; *n* (%)	32 (9.4)	24 [24 (11.2)]	8 (6.4)	0.14
Cancer; *n* (%)				
Without metastasis	5 (1.5)	4 (1.9)	1 (0.8)	0.54
With metastasis	1 (0.3)	1 (0.5)	0	
Severe COVID-19	114 (33.5)	35 (16.4)	79 (62.7)	<0.0001
Previous treatment				
Angiotensin Converting Enzyme Inhibitors; *n* (%)	48 (14.1)	20 (9.35)	28 (22.2)	0.001
Angiotensin Receptor Blocker; *n* (%)	66 (19.4)	47 (22)	19 (15.1)	0.12
Anticoagulants; *n* (%)	31 (9.1)	18 (8.4)	13 (10.4)	0.54
Antiplatelets; *n* (%)	48 (14.1)	26 (12.2)	22 (17.5)	0.17
Systemic glucocorticoids; *n* (%)	9 (2.7)	5 (2.3)	4 (3.2)	0.64
Immunosuppressants; *n* (%)	11 (3.2)	8 (3.7)	3 (2.4)	0.49

Of all patients, 126 (37.1%) had at least one positive SARS-CoV-2 viremia during the first week of hospitalization. Patients with viremia were more frequently male (72.2% vs. 54.2%, *p* = 0.001), dyslipidemic (45.2% vs. 34.6%, *p* = 0.05), had more severe disease (16.4% vs. 62.7%, *p* < 0.0001) and were more frequently treated with Angiotensin Converting Enzyme Inhibitors (ACEI) (22.2% vs. 9.4%, *p* = 0.001), as described in Table 1.

**Figure 1:**
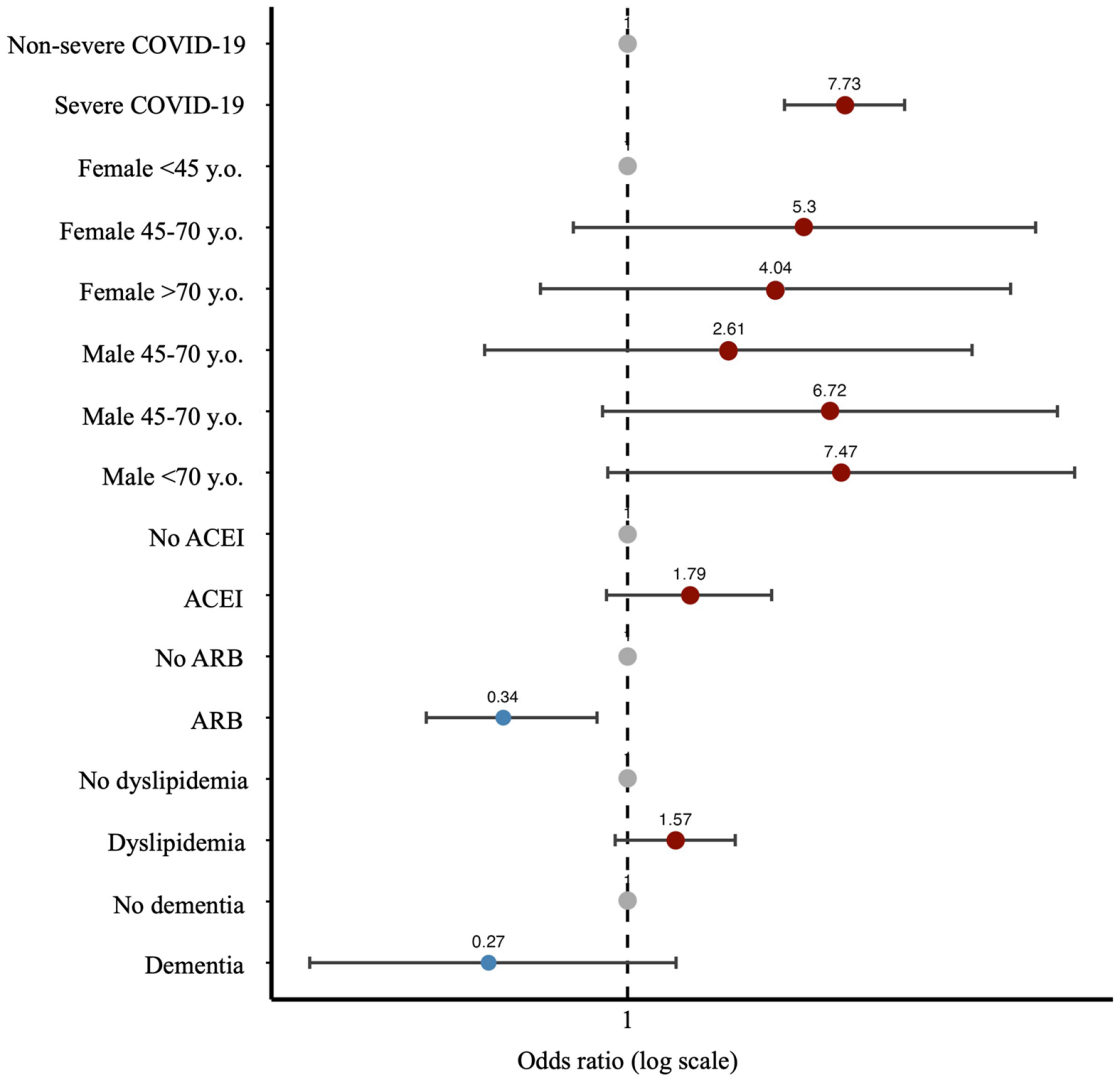
Clinical model. Forest plot with the Odds ratio and 95% Confidence Interval of each variable in the clinical model. Blue dots: protective effect against viremia. Red dots, favors viremia. ARB, Angiotensin II Receptor Blocker; ACEI, Angiotensin Converting Enzyme Inhibitor; y.o.: years old.

Multivariate analysis shown in Supplementary Table S6 and Figure 1 demonstrated that viremia was higher in males above 45 y-o compared to women younger than 45 y-o (OR 6.72 for 45 to 70y-o and OR 7.47 for >70 y-o; *p* = 0.08 and *p* = 0.07 respectively), in those with dyslipidemia [OR 1.57 (95%CI 0.89–2.76); *p* = 0.12], severe COVID-19 [OR 7.73 (95%CI 4.39–13.62); *p* < 0.0001], and those treated with ACEI (OR 1.79 [95%CI 0.82–3.89]; *p* = 0.14). By contrast, patients with dementia [OR 0.27 (95%CI 0.05–1.58); *p* = 0.147] and treatment with Angiotensin Receptor Blocker (ARB) [OR 0.34 (95%CI 0.34–0.7); *p* = 0.007] had viremia less frequently.

### 3.2. Genetic factors associated with viremia

Once the clinical model was established each of the 38 SNPs (9 from study A and 29 from study B) were individually included/forced in the model (Supplementary Table S7). Among them, 15 reached a value of *p*<0.15: rs33980500, rs13196377 and rs13190932 (*TRAF3IP2*), rs11052877 (*CD69*), rs2071746 (*HMOX1*), rs713400 (*TMPRSS2*), rs78958998 (*SERPING1*), rs541862 (*CFB*), rs438781 (*CFHR1*), rs12408446 (*CFHR3*), rs731034 (*COLEC11*), rs2660 (*OAS1*), rs280500 (*TYK2*), rs896 (*VIPR1*) and rs885863 (*VIPR2*). Of the three SNPs in *TRAF3IP2*, only rs33980500 was considered, as the three of them act in the same pathway and this SNP had the best performance. Also, rs43878 in *CFHR1* and rs12408446 in *CFHR3* were excluded because they had a high number of missing values. The rest of SNPs were included altogether with the clinical variables in order to determine interactions between them and also with clinical variables.

**Figure 2:**
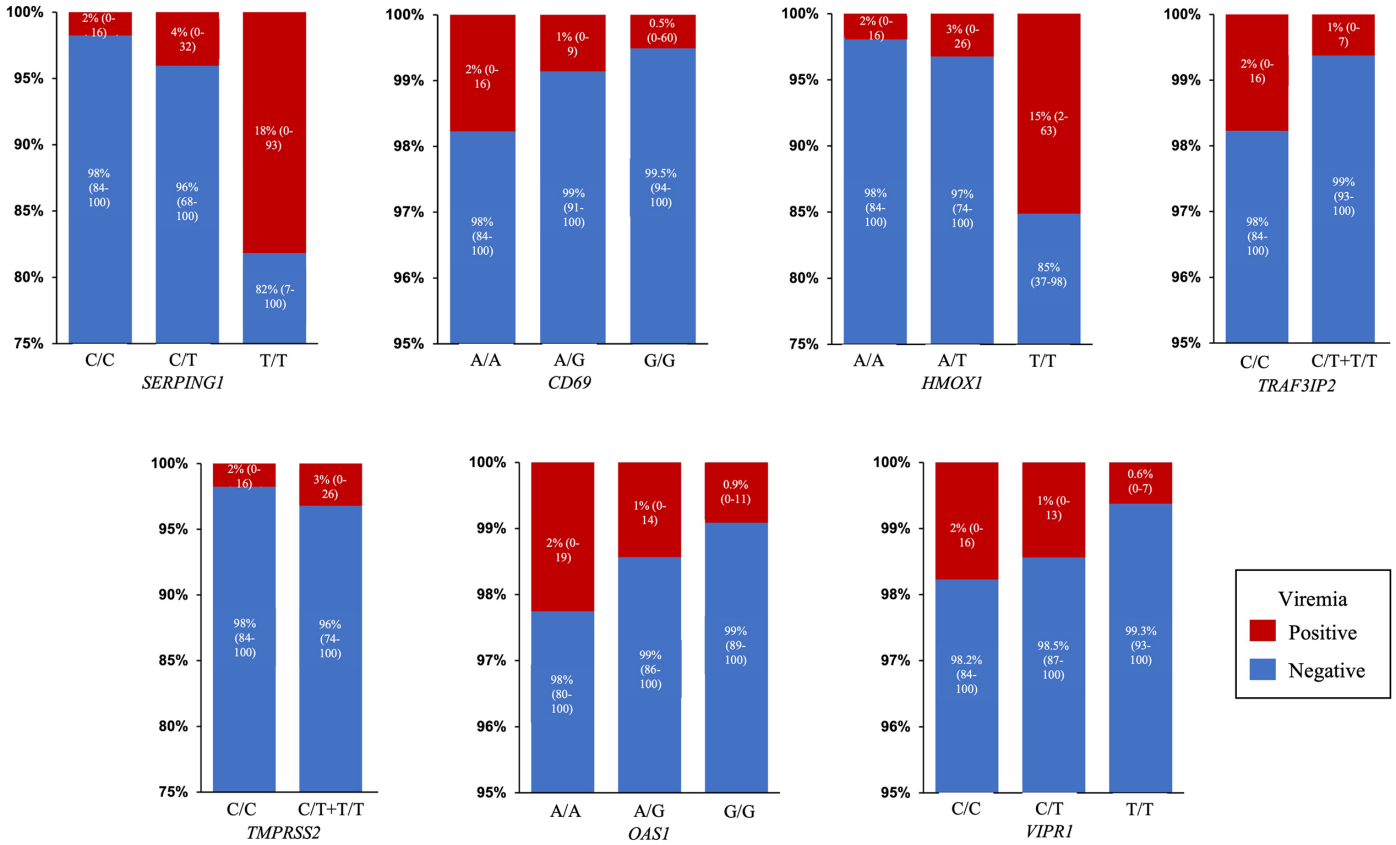
Predicted probability of viremia. Percentage and 95% Confidence Interval of predicted probability of viremia for each SNP genotype in the final model: *SERPING1* (rs78958998), *CD69* (rs11052877), *HMOX1* (rs2071746), *TRAF3IP2* (rs33980500), *TMPRSS2* (rs713400), *OAS1* (rs2660) and *VIPR1* (rs896).

Interestingly, in this last composite multivariate analysis (Table 2) most variables, especially the relationship with age and sex, improved their association with viremia both in terms of OR and value of p, except for rs713400 in *TMPRSS2* which slightly worsened. Thus, after adjustment by clinical and therapeutic variables the presence of the minor alleles of rs2071746 (*HMOX1*; T/T genotype OR 9.9 *p* < 0.0001), rs78958998 (probably associated with *SERPING1* expression; A/T genotype OR 2.3, *p* = 0.04 and T/T genotype OR 12.9, *p* < 0.0001), and rs713400 (eQTL for *TMPRSS2*; C/T + T/T genotype OR 1.86, *p* = 0.10) were associated with higher risk of viremia, whereas the minor alleles of rs11052877 (*CD69*; A/G genotype OR 0.5, *p* = 0.04 and G/G genotype OR 0.3, *p* = 0.01), rs2660 (*OAS1*; A/G genotype OR 0.6, *p* = 0.08), rs896 (*VIPR1*; T/T genotype OR 0.4, *p* = 0.02) and rs33980500 (*TRAF3IP2*; C/T + T/T genotype OR 0.3, *p* = 0.01) were associated with lower risk of viremia. The predicted probability of viremia per genotype of every significant SNP in this model is shown in Figure 2.

**Table 2:** Final model of variables predicting viremia.

	OR (95%CI)	*p* value
Age and sex (reference female <45 years)		
Female 45–70 years	21.99 (5.17–93.55)	<0.0001
Female >70 years	8.69 (1.94–38.99)	0.005
Male <45 years	6.06 (1.22–30.17)	0.03
Male 45–70 years	21.00 (5.62–78.54)	<0.0001
Male >70 years	11.02 (2.69–45.22)	0.001
Severe COVID-19	11.13 (5.27–23.50)	<0.0001
Angiotensin Converting Enzyme Inhibitors	2.40 (0.99–5.82)	0.052
Angiotensin II Receptor blocker	0.41 (0.16–1.10)	0.08
*CD69* rs11052877 (reference A/A)		
A/G	0.48 (0.24–0.96)	0.04
G/G	0.29 (0.11–0.74)	0.01
*HMOX1* rs2071746 (reference A/A)		
A/T	1.85 (0.86–3.99)	0.11
T/T	9.86 (3.42–28.42)	<0.0001
*SERPING1* rs78958998 (reference C/C)		
C/T	2.32 (1.02–5.28)	0.04
T/T	12.90 (3.91–42.63)	<0.0001
*TMPRSS2* rs713400 (reference C/C)		
C/T + T/T	1.86 (0.88–3.94)	0.10
*TRAF3IP2* rs33980500 (reference C/C)		
C/T + T/T	0.34 (0.15–0.78)	0.01
*OAS1* rs2660 (reference A/A)		
A/G	0.56 (0.29–1.07)	0.08
G/G	0.50 (0.13–1.99)	0.33
*VIPR1* rs896 (reference C/C)		
C/T	0.80 (0.40–1.60)	0.52
T/T	0.35 (0.15–0.83)	0.02

CI, Confidence Interval; OR, Odds Ratio.

## 4. Discussion

After 3 years of pandemic, COVID-19 remains as a very heterogeneous clinical picture with few reliable biomarkers for severity prediction at the beginning of disease. Among them, the presence of SARS-CoV-2 viremia seems to be the most solid (5). Although several genome wide analysis studies have been performed to find genetic variants associated with disease severity, to the best of our knowledge, this is the first study that has assessed the relationship between different genetic variants and SARS-CoV-2 viremia. Our results show than only one genetic variant related with SARS-CoV-2 replication (rs713400 for *TMPRSS2*), and four related with inflammation/immune regulation (rs33980500 for *TRAF3IP2,* rs11052877 for *CD69*, rs2071746 for *HMOX1* and rs78958998 for *SERPING1*) were associated with the presence of viremia.

These results were obtained under careful adjustment by several confounding variables previously suggested as factors associated with COVID-19 severity (3, 21). On the other hand, we must take into account that severity and viremia correlate. However, after adjusting our analysis by COVID-19 severity, the 7 SNPs described remained significant (except for rs713400 in *TMPRSS2* and rs2660 in *OAS1*), meaning that their association with viremia was independent of severity (Supplementary Table S8). This approach allowed us to realize that both genetic and clinical variables improved their performance when they were analyzed together, suggesting that the mechanisms leading to viremia and, therefore, COVID-19 severity involve complex interactions between genetic, sociodemographic, therapeutic and clinical factors. Furthermore, the most important variables to predict viremia seemed to be age and sex, supporting that, as in many other diseases, genetic background is made up of many items with a low contribution by each one (22, 23).

*TMPRSS2* encodes a transmembrane protease serine 2 involved in SARS-CoV-2 entry into host cells, by cleaving the spike (S) protein (24). rs713400 location in the 5’UTR of *TMPRSS2* could influence the expression of this gene (25). Our data indicate that carrying one copy of the T allele in rs713400 could be associated with higher prevalence of viremia, although after adjusting by COVID-19 severity this SNP was not significant (data not shown: *p* = 0.004 in a previous model without severity). Taking into account the role of *TMPRSS2* in viral entrance, this SNP could be associated with both viremia and severity. Thus, changes in *TMPRSS2* expression could modify the ability of SARS-CoV-2 to infect host cells and disseminate. In addition, several authors have assessed the influence of genetic variants of *TMPRSS2*, finding that some SNPs such as rs12329760 or rs75603675 are associated with COVID-19 severity (13, 15, 26–28).

Regarding immune system modulation, *TRAF3IP2* encodes for ACT1, a signaling adaptor involved in the regulation of IL-17-dependent immune responses and the activation of NF-κB (29). The variant rs33980500 is mainly associated with psoriasis and is located in a coding region of this gene, causing a change from aspartic to asparagine. Functional assays have found that this change causes a reduced binding of TRAF6 to ACT1, thereby leading to a decrease in IL-17 and Th17 responses (30, 31). In this regard, it has been proposed that Th17 cells play an important role in COVID-19 by promoting a proinflammatory immune response, with a correlation between intense Th17 responses and COVID-19 severity (32). Patients carrying the T allele in rs33980500 might have a weaker activation of IL-17-dependent proinflammatory pathways with a better viral control.

*CD69* also plays an important regulatory role in the immune system. CD69 deficient mice display more severe clinical pictures in the collagen induced arthritis and autoimmune myocarditis murine models (33, 34) and show an enhanced differentiation toward Th17 cells (35). In addition, in humans *CD69* expression is decreased in Treg cells from patients with systemic scleroderma (36) and response to tocilizumab is higher in rheumatoid arthritis patients homozygous for the mayor allele of rs11052877 (37). Here, we have described that patients carrying the minor allele of rs11052877 show a lower risk of SARS-CoV-2 viremia, in an additive fashion. In addition, we previously reported that patients with viremia show higher levels of IL-6 compared to those without viremia (38), and therefore, higher possibility of having a good response to tocilizumab (39). Although there are no studies on the role of rs11052877 in *CD69* expression, this SNP is located in the 3’ UTR which usually involves regulatory functions. Accordingly, it is tempting to propose that patients carrying the minor allele of rs11052877 could have higher levels of *CD69* expression, therefore leading to decreased Th17 responses to the virus allowing less inflammatory responses though with a better control of viral spreading.

*VIPR1* encodes the Vasoactive Intestinal Peptide (VIP) receptor type 1, called VPAC1. Through its binding to VPAC1 (constitutively expressed) o VPAC2 (inducible), VIP is involved in the anti-inflammatory response by promoting the expression of anti-inflammatory cytokines and inhibiting the production of pro-inflammatory cytokines such as TNF-∝ or IL-12 (40). In addition, VIP also plays a role in the regulation of Th cells, decreasing the profile of cytokines related to Th1 and Th17, inhibiting Th17 and its pathogenic phenotype (40). The rs896 in the 3’UTR of *VIPR1* has been shown to regulate the expression of VPAC1. The presence of the C allele has been associated with a decreased gene expression and an enhanced binding of the miRNA 525-5p, which decreases VPAC1 expression (41). This SNP has not been studied in COVID-19, but VIP levels were increased in patients with severe disease and correlated with lower levels of inflammatory biomarkers and survival of those patients (42). In this regard, we show that the T allele of rs896 was associated with lower risk of viremia, probably due to an increased expression of VPAC1 compared to C allele, promoting an anti-inflammatory response and the inhibition of Th17.

OAS1 (2′-5′ oligoadenylate synthetase 1) is part of the interferon I pathway and its main role is the activation of L RNAse, which is involved in the control of viral dissemination by degrading viral RNA (43). rs2660 in the 3’ UTR of *OAS1* has been previously associated with SARS-CoV infection, being the genotypes A/G and G/G protective (44). This SNP has also been studied in COVID-19, the study of Banday et al. found that the A allele entailed higher risk of hospitalization, as well as lower viral clearance efficiency (although this was not significant) (45). Probably these results are due to an increased enzymatic activity in OAS1 associated to the G/G genotype, the Neanderthal variant, compared to A/A genotype (46, 47). In our cohort, the A/G genotype had a tendency (*p* = 0.08) to be protective against viremia, which is consistent with the evidence described, as the G allele is associated with increased OAS1 activity and thus, viral clearance.

Another gene related to COVID-19 pathogenesis is *HMOX1*, which encodes heme oxygenase one (HO-1), a protein involved in heme catabolism with anti-inflammatory effects (48). HO-1 levels are associated with acute respiratory distress syndrome (ARDS) (49, 50), as well as with COVID-19 severity (51, 52) and this gene has been proposed as a therapeutic target for this disease (53–55). The SNP rs2071746 has not been linked specifically to COVID-19 but Ono et al. showed that the A allele increased *HMOX1* promoter activity compared to the T allele (56, 57). This fact could lead to a protective anti-inflammatory and antiviral effect of the A allele by increasing the expression of IL-10 and the interferon signaling pathway as well as promoting the switch to anti-inflammatory M2 macrophages (58–60). This fits well with our observation that patients homozygous for the T allele of rs2071746 show higher levels of viremia.

Finally, and also in accordance with the notion that excessive inflammatory responses can be associated with lower capability to control SARS-CoV-2 spreading, rs78958998 has been described as an eQTL for *SERPING1*, and one study suggested its association with COVID-19 (61). *SERPING1* encodes the protein C1 inhibitor (C1INH) which is involved in complement and coagulation pathways as well as contact system by inhibiting C1r and C1s or activated factor XI and XII, among others (62). Although C1INH levels are increased in patients with COVID-19, it might be insufficient to control thromboinflammation. Reasons for this include a relative deficiency due to an uncontrolled activation of complement and coagulation cascades, together with the limitation of its regulatory activity caused by the interaction with SARS-CoV-2 proteins (63, 64). Since complement activation is involved in virus neutralization and virolysis, impaired *SERPING1* expression could contribute to virus dissemination and viremia (65).

Although the implication of the variants presented in this manuscript in the prevalence of viremia is attractive and based on the function of each of the genes, many of the SNPs described above have not been studied in COVID-19 patients. In addition, functional studies are needed to correlate these variants with their gene expression and protein activity.

The main limitation of this study is the small sample size, which was affected by the previous studies of our group. However, this sample size was enough to find significant differences in those SNPs with the strongest effect. Obviously, a wider approach in terms of genetic variations would be desirable; however, the study of a higher number of genes was precluded by two issues, the high economic cost of these studies, and the need of a larger number of patients. Another important limitation is the lack of data about SARS-CoV-2 variants and vaccination status, which could differentially affect infectivity and prevalence of viremia. However, most of the patients suffered from COVID-19 between the first and the fourth waves of the pandemic, so the effect of vaccination could be considered minor.

In conclusion, SARS-CoV-2 viremia was associated with variants of rs2071746 (*HMOX1*) rs78958998 (*SERPING1*), rs713400 (*TMPRSS2*), rs11052877 (*CD69*), rs33980500 (*TRAF3IP2*), rs2660 (*OAS1*) and rs896 (*VIPR1*), after adjusting by age and sex, COVID-19 severity and treatment with ACE inhibitors and Angiotensin II blockers. Nevertheless, these results should be validated in a different cohort.

## Data availability statement

The datasets presented in this study can be found in online repositories. The names of the repository/repositories and accession number (s) can be found at: https://doi.org/10.5281/zenodo.7882217.

## Ethics statement

The studies involving humans were approved by Research Ethics Committee of University Hospital La Princesa, Madrid. The studies were conducted in accordance with the local legislation and institutional requirements. The ethics committee/institutional review board waived the requirement of written informed consent for participation from the participants or the participants' legal guardians/next of kin because due to the COVID-19 pandemic emergency, oral consent was accepted as proposed by the AEMPS (Agencia Española de Medicamentos y Productos Sanitarios, The Spanish Agency for Medicines and Medical Devices).

## Author contributions

ER-V, LC, and IG-Á designed the study and wrote the first draft of the manuscript. ER-V, AL, JG-R, GVG, PZ, MC, LR, MS, CR, AV, JR, CM, JH, FA, IS, DR, RG-V, CSF, and RPG included patients in the study and collected data. RC-R, SC-O, PD-W, AM-J, CM-C, and EF-R extracted and processed samples. ER-V, SC-O, PD-W, AT-M, and NZ-C performed laboratory determinations. ER-V, NM, and IG-Á analyzed data. ER-V, PZ, SC-O, PD-W, AT-M, NM, RC-R, NZ-C, AM-J, AL, JG-R, GVG, AV, JR, CM, JH, PD-F, FA, IS, DR, RG-V, CSF, EF-R, IG-Á, and LC reviewed the final draft. All authors contributed to the article and approved the submitted version.

## Group members of PREDINMUN-COVID group

Internal Medicine-Infectious Diseases: Ana Barrios, Jesús Sanz, Pedro Casado, Ángela Gutiérrez, Azucena Bautista, Pilar Hernández, Nuria Ruiz Giménez, Berta Moyano, Paloma Gil, María Jesús Delgado, Pedro Parra, Beatriz Sánchez, Carmen Sáez, Marta Fernández Rico, Cristina Arévalo Román; Rheumatology: Santos Castañeda, Irene Llorente, Eva G. Tomero, Noelia García Castañeda, Miren Uriarte; Microbiology: Leticia Fontán García-Rodrigo, Diego Domingo García, Teresa Alarcón Cavero, María Auxiliadora Semiglia Chong, Ainhoa Gutiérrez Cobos; Immunology: Francisco Sánchez-Madrid, Enrique Martín Gayo, Ildefonso Sánchez-Cerrillo, Pedro Martínez-Fleta, Celia López-Sanz, Ligia Gabrie, Luciana del Campo Guerola, Reyes Tejedor; Pneumology: Julio Ancochea, Elena García Castillo, Elena Ávalos, Ana Sánchez-Azofra, Tamara Alonso, Carolina Cisneros, Claudia Valenzuela, Francisco Javier García Pérez, Rosa María Girón, Javier Aspa, Celeste Marcos, M. del Perpetuo Socorro Churruca, Enrique Zamora, Adrián Martínez, Mar Barrio Mayo, Rosalina Henares Espi; Anesthesiology: Rosa Méndez, David Arribas; Intensive Care Unit: Marta Chicot Llano, Begoña González, Begoña Quicios, Pablo Patiño, Marina Trigueros, Cristina Dominguez Peña, David Jiménez Jiménez, Pablo Villamayor, Alfonso Canabal; Hematology: Rafael de la Cámara, Javier Ortiz, Isabel Iturrate.

## Funding

This study was funded with grants: “Fondos Supera COVID19” by Banco Santander and CRUE to CSF, RG-V, CM-C, and RPG; RD21/0002/0027, PI18/0371 and PI21/00526 to IG-Á, and PI19/00096 to EF-R from Ministerio de Economía y Competitividad (Instituto de Salud Carlos III) and co-funded by European regional development fund (ERDF) “A way to make Europe”; and co-financed by the Community of Madrid through the Covid 2019 Aid. The work of ER-V has been funded by a Rio-Hortega grant CM19/00149 from the Ministerio de Economía y Competitividad (Instituto de Salud Carlos III) and co-funded by The European Regional Development Fund (ERDF) “A way to make Europe.” GVG is co-financed by Instituto de Salud Carlos III (ISCIII) and the European Social Fund (PFIS predoctoral grant, number FI20/00090). PZ is financed by Universidad Autónoma de Madrid, Margarita Salas contract, grants for the requalification of the Spanish university system. None of these sponsors have had any role in study design; in the collection, analysis, and interpretation of data; in the writing of the report; and in the decision to submit the article for publication.

## Conflict of interest

FA, has been consultant or investigator in clinical trials sponsored by the following pharmaceutical companies: Abbott, Alter, Aptatargets, Chemo, FAES, Farmalíder, Ferrer, Galenicum, GlaxoSmithKline, Gilead, Italfarmaco, Janssen-Cilag, Kern, Normon, Novartis, Servier, Teva and Zambon. IG-Á reports grants from Instituto de Salud Carlos III, during the course of the study; personal fees from Lilly and Sanofi; personal fees and non-financial support from BMS; personal fees and non-financial support from Abbvie; research support, personal fees and non-financial support from Roche Laboratories; research support from Gebro Pharma; non-financial support from MSD, Pfizer and Novartis, not related to the submitted work.

The remaining authors declare that the research was conducted in the absence of any commercial or financial relationships that could be construed as a potential conflict of interest.

## Publisher’s note

All claims expressed in this article are solely those of the authors and do not necessarily represent those of their affiliated organizations, or those of the publisher, the editors and the reviewers. Any product that may be evaluated in this article, or claim that may be made by its manufacturer, is not guaranteed or endorsed by the publisher.
