# Supplementary material for: Occurrence of SARS-CoV-2 viremia is associated with genetic variants of genes related to COVID-19 pathogenesis
Source: Front Med (Lausanne). 2023 Sep 22;10:1215246. doi: 10.3389/fmed.2023.1215246 (PMC10557488; doi:10.3389/fmed.2023.1215246)
Supplement: Supplementary file 1 [file Data_Sheet_1.docx]

Supplementary Material

Occurrence of SARS-CoV-2 viremia is associated with genetic variants of genes related to COVID-19 pathogenesis

**Authors**: Emilia Roy-Vallejo^1,2^*, Sara Fernández de Córdoba-Oñate^3^, Pablo Delgado-Wicke^4^, Ana Triguero-Martínez^2,3^, Nuria Montes Casado^2,3^, Rosa Carracedo-Rodríguez^4^, Nelly Zurita-Cruz^5^, Ana Marcos-Jiménez^2,6^, Amalia Lamana^7^, José María Galván-Román^1,2^, Gonzalo Villapalos^2,8^, Pablo Zubiaur^2,8^, Marianela Ciudad^1^, Laura Rabes^1^, Marta Sanz^1^, Carlos Rodríguez^1^, Almudena Villa^1^, Jesús Álvarez Rodríguez^1^, Celeste Marcos^9^, Julia Hernando^10^, Francisco Abad^2,8,11^, Ignacio de los Santos^1,2,12^, Diego A. Rodríguez Serrano^13^, Rosario García-Vicuña^2,3^, Carmen Suárez Fernández^1,2,14^, Rosa P. Gomariz^7^, Cecilia Muñoz-Calleja^2,6,14^, Elena Fernández-Ruiz^2,4^, Isidoro González Álvaro^2,3¶^ and Laura Cardeñoso^2,5¶^, on behalf of the PREDINMUN-COVID Group

*** Correspondence:** eroyvallejo@gmail.com

# Supplementary Tables

Supplementary Table 1. Genetic variants included in study A.

| Gene | SNP (rs) |
| --- | --- |
| *ABCB1* | rs1045642 |
|  | rs1128503 |
|  | rs2032582 |
|  | rs2032582 |
| *ABO* | rs657152 |
| *ACE* | rs4291 |
|  | rs1799752 |
|  | rs4343 |
| *ACE2* | rs143695310 |
|  | rs2106809 |
|  | rs1978124 |
|  | rs5936029 |
|  | rs1996225 |
|  | rs4646156 |
|  | rs2285666 |
|  | rs2074192 |
|  | rs35803318 |
|  | rs4830542 |
|  | rs4646116 |
|  | rs4646188 |
|  | rs41303171 |
| *ADAM17* | rs55790676 |
|  | rs12692386 |
| *AGT* | rs699 |
| *ApoE* | rs7412 |
|  | rs429358 |
| *CCL2* | rs1024611 |
| *CCL5* | rs2107538 |
| *CD14* | rs2569190 |
| *CD147* | rs8259 |
| *CD69* | rs11052877 |
| *CLEC2D* | rs1560011 |
| *CRP* | rs1130864 |
| *CSF3* | rs2227322 |
| *CXCL1* | rs2071425 |
| *CYP2C19* | rs12248560 |
|  | rs4244285 |
| *CYP2C9* | rs1799853 |
|  | rs1057910 |
| *CYP3A4* | rs67666821 |
|  | rs35599367 |
| *CYP3A5* | rs776746 |
| *CYP4V2* | rs13146272 |
| *DPP4* | rs56179129 |
|  | rs116302758 |
|  | rs17574 |
|  | rs17848916 |
| *ENOX1* | rs9594987 |
| *EPHX1* | rs1051740 |
| *F11* | rs2289252 |
|  | rs2036914 |
| *FGG* | rs2066865 |
| *G6PD* | rs1050828 |
|  | rs1050829 |
|  | rs5030868 |
| *GC (DBP)* | rs7041 |
|  | rs4588 |
| *HCP5* | rs2395029 |
| *HMOX-1* | rs2071746 |
| *IFITM3* | rs12252 |
| *IFNL3* | rs12979860 |
| *IL10* | rs1800896 |
|  | rs1800871 |
| *IL13* | rs1800925 |
| *IL17A* | rs3819025 |
|  | rs2275913 |
| *IL1B* | rs1143627 |
|  | rs1143634 |
| *IL1RN* | rs315952 |
| *IL6* | rs1800795 |
|  | rs1800796 |
|  | rs1818879 |
| *IL6R* | rs2228145 |
|  | rs4329505 |
|  | rs7529229 |
|  | rs11265618 |
|  | rs12083537 |
| *INFL4* | rs11322783 |
| *Intergenic region* | rs10108210 |
|  | rs703297 |
| *KCNMB1* | rs703505 |
| *LZTFL1* | rs35044562 |
| *MTHFR* | rs1801131 |
|  | rs1801133 |
| *NFKB* | rs28362491 |
| *NLRP3* | rs10754555 |
| *PEAR1* | rs12041331 |
| *PTGS1* | rs10306114 |
| *SLCO1B1* | rs4149056 |
| *TLR1* | rs5743551 |
| *TLR2* | rs1898830 |
|  | rs7656411 |
|  | rs11938228 |
|  | rs3804099 |
|  | rs1816702 |
| *TLR4* | rs1927911 |
|  | rs5030728 |
| *TLR9* | rs187084 |
|  | rs352162 |
| *TMPRSS2* | rs55964536 |
|  | rs383510 |
|  | rs464397 |
|  | rs463727 |
|  | rs713400 |
|  | rs8134378 |
|  | rs469390 |
|  | rs734056 |
|  | rs2070788 |
|  | rs12329760 |
|  | rs77675406 |
|  | rs75603675 |
| *TNF* | rs1800610 |
|  | rs1799964 |
|  | rs361525 |
| *TNF/TNKA* | rs1800629 |
| *TNKA* | rs1800630 |
| *TRAF3IP2* | rs13190932 |
|  | rs33980500 |
|  | rs13196377 |
| *VDR* | rs2228570 |

SNP: Single Nucleotide Polymorphism

Supplementary table 2. Genetic variants included in study B.

| Gene | SNP (rs) |
| --- | --- |
| *CFB* | rs541862 |
| *CFH* | rs800292 |
| *CFHR1* | rs438781 |
| *CFHR3* | rs12408446 |
| *COLEC11* | rs731034 |
| *C3* | rs2230199 |
| *GGCX* | rs12714145 |
| *OAS1* | rs1131454  rs2660 |
| *SERPING1* | rs78958998 |
| *SOCS1* | rs4780355 |
| *STAT1* | rs13005843 |
| *STAT4* | rs7574865 |
| *TLR7* | rs179008  rs179010  rs3853839 |
| *TYK2* | rs12720270  rs2304256  rs280500  rs280519  rs8108236  rs34536443  rs12720356 |
| *VIP* | rs35643203  rs688136 |
| *VIPR1* | rs7628235  rs896 |
| *VIPR2* | rs399867  rs885863 |

SNP: Single Nucleotide Polymorphism

Supplementary Table 3. Genetic variants included in the study

| Gene | SNP (rs) | MAF (Global) | Impact variant | Taqman Probe Part Number |
| --- | --- | --- | --- | --- |
| *CD69* | rs11052877 | 0.37 (G) | 3’ UTR variant | [C__32169538_10](https://www.thermofisher.com/order/genome-database/details/genotyping/C__32169538_10?CID=&ICID=&subtype=) |
| *CFB* | rs541862 | 0.09 (C) | Intron variant | C__940292_10 |
| *CFH* | rs800292 | 0.25 (A) | Missense variant | C__2530382_10 |
| *CFHR1* | rs438781 | 0.48 (A) | Intron variant | C__26010839_20 |
| *CFHR3* | rs12408446 | 0.21 (A) | Unknown | C__2521730_10 |
| *COLEC11* | rs731034 | 0.14 (C) | Intron variant | C__1014547_10 |
| *CXCL1* | rs2071425 | 0.20 (G) | Synonymous variant | [C__22274655_10](https://www.thermofisher.com/order/genome-database/details/genotyping/C__22274655_10?CID=&ICID=&subtype=) |
| *C3* | rs2230199 | 0.20 (C) | Missense variant | C__26330755_10 |
| *GGCX* | rs12714145 | 0.40 (T) | Intron variant | C__31839079_10 |
| *HMOX1* | rs2071746 | 0.46 (T) | 2KB Upstream variant | [C__15869717_10](https://www.thermofisher.com/order/genome-database/details/genotyping/C__15869717_10?CID=&ICID=&subtype=) |
| *OAS1* | rs1131454  rs2660 | 0.44 (G)  0.34 (G) | Missense variant  Stop gained | C__2567435_20  C__2567429_10 |
| *SERPING1* | rs78958998 | 0.10 (T) | 500B Downstream Variant | C__105427933_10 |
| *SOCS1* | rs4780355 | 0.33 (C) | 500B Downstream Variant | C__3189846_20 |
| *STAT1* | rs13005843 | 0.06 (T) | Intron variant | C__31155526_10 |
| *STAT4* | rs7574865 | 0.23 (T) | Intron variant | C__29882391_10 |
| *TLR7* | rs179008  rs179010  rs3853839 | 0.20 (T)  0.30 (T)  0.18 (G) | Missense variant  Intron variant  3’ UTR variant | C__2259574_10  C__2259576_10  C__2259573_10 |
| *TMPRSS2* | rs75603675  rs713400 | 0.30 (A)  0.11 (T) | Missense variant  Unknown | C__102710002_10  C__824609_10 |
| *TNF* | rs1800629 | 0.15 (A) | 2KB Upstream variant | C__7514879_10 |
| *TRAF3IP2* | rs13196377  rs33980500  rs13190932 | 0.05 (A)  0.07 (T)  0.06 (A) | Intron variant  Missense variant  Missense variant | C__2475647_10  C__2473124_10  C___2473123_20 |
| *TYK2* | rs12720270  rs2304256  rs280500  rs280519  rs8108236  rs34536443  rs12720356 | 0.16 (A)  0.28 (A)  0.16 (G)  0.50 (A)  0.09 (A)  0.04 (C)  0.08 (C) | Intron variant  Missense variant  Non coding transcript variant  Intron variant  Intron variant  Missense variant  Missense variant | C__1931075_10  C__25473911_10  C__944731_10  C__944724_30  C__34043032_10  C__60866522_10  C__34042925_10 |
| *VIP* | rs35643203  rs688136 | 0.04 (T)  0.38 (C) | Intron variant  3’ UTR variant | C__3250637_10  C__3250639_10 |
| *VIPR1* | rs7628235  rs896 | 0.01 (G)  0.39 (T) | Intron variant  Non coding transcript variant | C__189407884_10  C__3033184_10 |
| *VIPR2* | rs399867  rs885863 | 0.44 (G)  0.46 (C) | Intron variant  Non coding transcript variant | C__3266365_20  C__7556951_20 |

SNP: Single Nucleotide Polymorphism; MAF: Minor Allele Frequency; UTR: Untranslated Region

Supplementary table 4. Bivariate and multivariate logistic regression analysis of clinical variables

| Variable | Bivariate analysis | | Multivariate analysis* | |
| --- | --- | --- | --- | --- |
|  | OR (95%CI) | p value | OR (95%CI) | p value |
| Male sex | 2.20 (1.37-3.52) | 0.001 |  |  |
| Age (categorized, reference <45)  - 45-70  - >70 | 4.94 (2.13-11.40)  3.07 (1.29-7.24) | 0.0001  0.01 |  |  |
| **Interaction of sex and age** (reference women <45)**  - Women 45-70  - Women >70  - Male <45  - Male 45-70  - Male >70 |  |  | 5.55 (0.61-50.86)  3.80 (0.40-36.36)  2.97 (0.29-30.63)  6.56 (0.73-58.93)  7.35 (0.77-70.34) | 0.13  0.25  0.36  0.09  0.08 |
| Race/ethnicity (reference White, non-Hispanic)  - White, Hispanic  - Afro-descendant  - Asian | 0.53 (0.29-0.97)  0  3.1 (0.65-∝) | 0.04  0.422  0.18 | 0.61 (0.28-1.30)  1  4.68 (0.48-45.98) | 0.20  0.19 |
| Hypertension | 1.31 (0.84-2.05) | 0.23 |  |  |
| Obesity | 1 (0.55-1.81) | 1 |  |  |
| **Dyslipidemia** | 1.56 (1-2.45) | 0.05 | 1.59 (0.89-2.83) | 0.12 |
| Myocardial infarction | 1.33 (0.58-3.07) | 0.51 |  |  |
| Heart failure | 0.77 (0.30-2.02) | 0.61 |  |  |
| **Dementia** | 0.27 (0-1.1) | 0.07 | 0.27 (0.04-1.57) | 0.14 |
| COPD | 0.54 (0.24-1.21) | 0.14 | 0.53 (0.20-1.42) | 0.21 |
| Diabetes mellitus | 1.41 (0.78-2.56) | 0.25 |  |  |
| Chronic Kidney Disease | 0.61 (0.20-1.84) | 0.39 |  |  |
| Cancer  - Without metastasis  - With metastasis | 0.42 (0-2.82)  0 | 0.42  0.44 |  |  |
| **Severe COVID-19** | 8.64 (5.19-14.39) | <0.0001 | 7.80 (4.39-13.86) | <0.0001 |
| **ACEI treatment** | 2.77 (1.50-5.14) | 0.001 | 1.82 (0.83-3.99) | 0.14 |
| **ARB treatment** | 0.63 (0.35-1.13) | 0.12 | 0.36 (0.16-0.82) | 0.02 |
| Previous corticosteroids | 1.37 (0.39-4.81) | 0.64 |  |  |

ACEI: Angiotensin Converting Enzyme Inhibitors; ARB: Angiotensin Receptor Blockers; CI: confidence interval; COPD: Chronic obstructive pulmonary disease; OR: odds ratio

* Variables with p<0.15 were included in the multivariate analysis. The final clinical model (in bold) was reached through backward stepwise removal of variables with p value>0.15.

**The interaction of sex and age was included in the multivariate analysis considering the different distribution of viremia by age depending on sex. It was based on the observations from a previous study (doi: 10.3389/fmed.2022.855639) and the current one.

Supplementary table 5. Treatment and laboratory data by viremia status

|  | Study population (n= 340) | No viremia (n = 214) | Viremia (n = 126) | p value |
| --- | --- | --- | --- | --- |
| ** Treatment during hospitalization |  |  |  |  |
| Hydroxicloroquine; n (%) | 117 (34.4) | 54 (25.2) | 63 (50) | <0.0001 |
| Lopinavir/ritonavir; n (%) | 100 (29.4) | 48 (22.4) | 52 (41.3) | <0.0001 |
| Glucocorticoids: n (%) | 300 (88.2) | 183 (85.5) | 117 (92.9) | 0.04 |
| Tocilizumab; n (%) | 111 (32.7) | 46 (21.5) | 65 (51.6) | <0.0001 |
| Remdesivir; n (%) | 22 (6.5) | 11 (5.1) | 11 (8.7) | 0.2 |
| Heparin; n (%) | 318 (93.5) | 202 (94.4) | 116 (92.1) | 0.4 |
| ** Laboratory findings |  |  |  |  |
| Lymphocytes/mm3; median (IQR)  NR: 1.00 -4.00 | 920 (635-1331) | 1045 (720-1460) | 755 (580-1040) | <0.0001 |
| Fibrinogen (mg/dl); median (IQR)  NR: 150-400 | 704 (596-807) | 697 (584-785) | 728 (613-823) | 0.1 |
| LDH (U/L); median (IQR)  NR: 135 - 225 | 298 (224-392) | 264 (208-340) | 380 (297-488) | <0.0001 |
| Serum IL-6 (pg/ml); median (IQR)  NR: < 30 pg/ml | 12 (3-32) | 9 (2-26.5) | 17.8 (7.9-64.4) | <0.0001 |
| Ferritin (ng/ml); median (IQR)  NR: 30 - 400 | 702 (356-1533) | 539 (303-1349) | 1158 (517-2097) | <0.0001 |
| CRP (mg/dL); median (IQR)  NR: 0.00 - 0.50 | 7.4 (3.6-14.1) | 5.9 (3.2-11) | 12 (7-20) | <0.0001 |
| D-dimer (µg/ml); median (IQR)  NR:0.14 - 0.50 | 0.7 (0.4-1.1) | 0.6 (0.4-1.1) | 0.7 (0.4-1.3) | 0.1 |

CRP: C-Reactive Protein; IQR: Interquartile Range; LDH: Lactate Dehydrogenase; NR: normal range

Supplementary Table 6. Clinical variables predicting the presence of SARS-CoV-2 viremia

|  | OR (95%CI) | p value |
| --- | --- | --- |
| Age and sex (reference female <45 years)   - Female 45-70 years - Female >70 years - Male <45 years - Male 45-70 years - Male >70 years | 5.30 (0.60-46.84)  4.04 (0.44-36.99)  2.61 (0.26-25.72)  6.72 (0.79-57.51)  7.47 (0.83-67.66) | 0.13  0.22  0.41  0.08  0.07 |
| Dyslipidemia | 1.57 (0.89-2.76) | 0.12 |
| Dementia | 0.27 (0.05-1.58) | 0.147 |
| Severe COVID-19 | 7.73 (4.39-13.62) | <0.0001 |
| Angiotensin Converting Enzyme Inhibitors | 1.79 (0.82-3.89) | 0.14 |
| Angiotensin Receptor Blocker | 0.34 (0.15-0.75) | 0.007 |

CI: Confidence Interval; OR: Odds Ratio

Supplementary table 7. Multivariate analysis including variables in the clinical model and each SNP

|  | OR (95%CI) | p value |
| --- | --- | --- |
| *CD69* rs11052877 (reference A/A)   - A/G - G/G | 0.60 (0.34-1.07)  0.38 (0.15-0.92) | 0.08  0.03 |
| *CFB* rs541862 (reference T/T)   - C/T - C/C | 1.67 (0.87-3.22)  2.53 (0.46-13.99) | 0.12  0.29 |
| *CFH* rs800292 (reference G/G)   - A/G - A/A | 1.28 (0.73-2.23)  0.43 (0.13-1.39) | 0.39  0.16 |
| *CFHR1* rs438781 (reference T/T)   - T/A - A/A | 1.07 (0.54-2.12)  2.21 (1.11-4.39) | 0.84  0.024 |
| *CFHR3* rs12408446 (reference G/G)   - G/A - A/A | 1.27 (0.68-2.36)  2.37 (1.06-5.30) | 0.46  0.04 |
| *COLEC11* rs731034 (reference T/T)   - C/T - T/T | 1.76 (0.93-3.34)  0.75 (0.07-8.62) | 0.09  0.82 |
| *CXCL1* rs2071425 (reference A/A)   - A/G - G/G | 1.31 (0.73-2.34)  1.02 (0.40-2.59) | 0.37  0.97 |
| *C3* rs2230199 (reference G/G)   - C/G - C/C | 0.97 (0.55-1.72)  0.58 (0.12-2.79) | 0.93  0.5 |
| *GGCX* rs12714145 (reference C/C)   - C/T - T/T | 0.97 (0.54-1.73)  1.71 (0.78-3.75) | 0.91  0.18 |
| *HMOX1* rs2071746 (reference A/A)   - A/T - T/T | 1.72 (0.93-3.20)  7.02 (2.94-16.74) | 0.09  <0.0001 |
| *OAS1* rs1131454 (reference A/A)   - A/G - G/G | 1.18 (0.49-2.83)  1.27 (0.52-3.10) | 0.71  0.61 |
| *OAS1* rs2660 (reference A/A)   - A/G - G/G | 0.62 (0.35-1.08)  0.62 (0.20-1.88) | 0.09  0.40 |
| *SERPING1* rs78958998 (reference C/C)   - C/T - T/T | 1.79 (0.89-3.62)  2.10 (0.08-53.05) | 0.10  0.65 |
| *SOCS1* rs4780355 (reference T/T)   - T/C - C/C | 1.27 (0.70-2.32)  0.81 (0.36-1.81) | 0.43  0.61 |
| *STAT1* rs13005843 (reference C/C)   - C/T - T/T | 0.89 (0.40-2.00)  Empty | 0.78 |
| *STAT4* rs7574865 (reference G/G)   - G/T - T/T | 1.02 (0.58-1.78)  1.45 (0.47-4.53) | 0.96  0.52 |
| *TLR7* rs179008 (reference A/A)   - T/A - T/T | 1.25 (0.48-3.29)  1.24 (0.60-2.57) | 0.65  0.56 |
| *TLR7* rs179010 (reference C/C)   - C/T - T/T | 0.97 (0.38-2.49)  1.39 (0.69-2.78) | 0.95  0.36 |
| *TLR7* rs3853839 (reference C/C)   - C/G - G/G | 0.99 (0.42-2.37)  0.81 (0.39-1.66) | 0.99  0.56 |
| *TMPRSS2* rs75603675 (reference C/C)   - A/C - A/A | 0.80 (0.45-1.41)  0.75 (0.33-1.71) | 0.44  0.49 |
| *TMPRSS2* rs713400 (reference C/C)   - C/T + T/T | 1.78 (0.95-3.33) | 0.07 |
| *TNF* rs1800629 (reference G/G)   - A/G - A/A | 1.08 (0.55-2.1)  0.22 (0.02-2.68) | 0.83  0.23 |
| *TRAF3IP2* rs13196377 (reference G/G)   - A/G - A/A | 0.51 (0.23-1.14)  Empty | 0.10 |
| *TRAF3IP2* rs33980500 (reference C/C)   - C/T + T/T | 0.40 (0.19-0.87) | 0.02 |
| *TRAF3IP2* rs13190932 (reference G/G)   - A/G - A/A | 0.46 (0.20-1.06)  Empty | 0.07 |
| *TYK2* rs12720270 (reference G/G)   - A/G - A/A | 1.00 (0.55-1.81)  0.43 (0.07-2.49) | 1  0.35 |
| *TYK2* rs2304256 (reference G/G)   - A/G - A/A | 0.92 (0.52-1.63)  0.57 (0.16-2.01) | 0.78  0.38 |
| *TYK2* rs280500 (reference A/A)   - A/G - G/G | 0.62 (0.33-1.17)  2.01 (0.38-10.77) | 0.14  1 |
| *TYK2* rs280519 (reference A/A)   - A/G - G/G | 0.83 (0.46-1.50)  0.67 (0.32-1.42) | 0.53  0.30 |
| *TYK2* rs8108236 (reference G/G)   - A/G - A/A | 0.56 (0.26-1.23)  Empty | 0.15 |
| *TYK2* rs34536443 (reference G/G)   - G/C - C/C | 2.16 (0.63-7.34)  Empty | 0.22 |
| *TYK2* rs12720356 (reference A/A)   - A/C - C/C | 0.72 (0.25-2.11)  Empty | 0.56 |
| *VIP* rs35643203 (reference C/C)   - C/T - T/T | 0.82 (0.37-1.81)  Empty | 0.62 |
| *VIP* rs688136 (reference T/T)   - C/T - C/C | 1.27 (0.72-2.22)  1.12 (0.46-2.76) | 0.41  0.80 |
| *VIPR1* rs7628235 (reference C/C)   - C/G - G/G | 1.25 (0.32-4.87)  Empty | 0.75 |
| *VIPR1* rs896 (reference C/C)   - C/T - T/T | 0.73 (0.40-1.31)  0.43 (0.20-0.94) | 0.28  0.04 |
| *VIPR2* rs399867 (reference A/A)   - A/G - G/FG | 1.19 (0.60-2.35)  0.96 (0.46-2.02) | 0.62  0.92 |
| *VIPR2* rs885863 (reference C/C)   - C/T - T/T | 0.71 (0.38-1.34)  0.50 (0.24-1.02) | 0.29  0.06 |

CI: Confidence Interval; OR: Odds Ratio

*Adjusted by the interaction of age and sex, dyslipidemia, dementia and ARB and ACEI treatment.

## Supplementary Figures


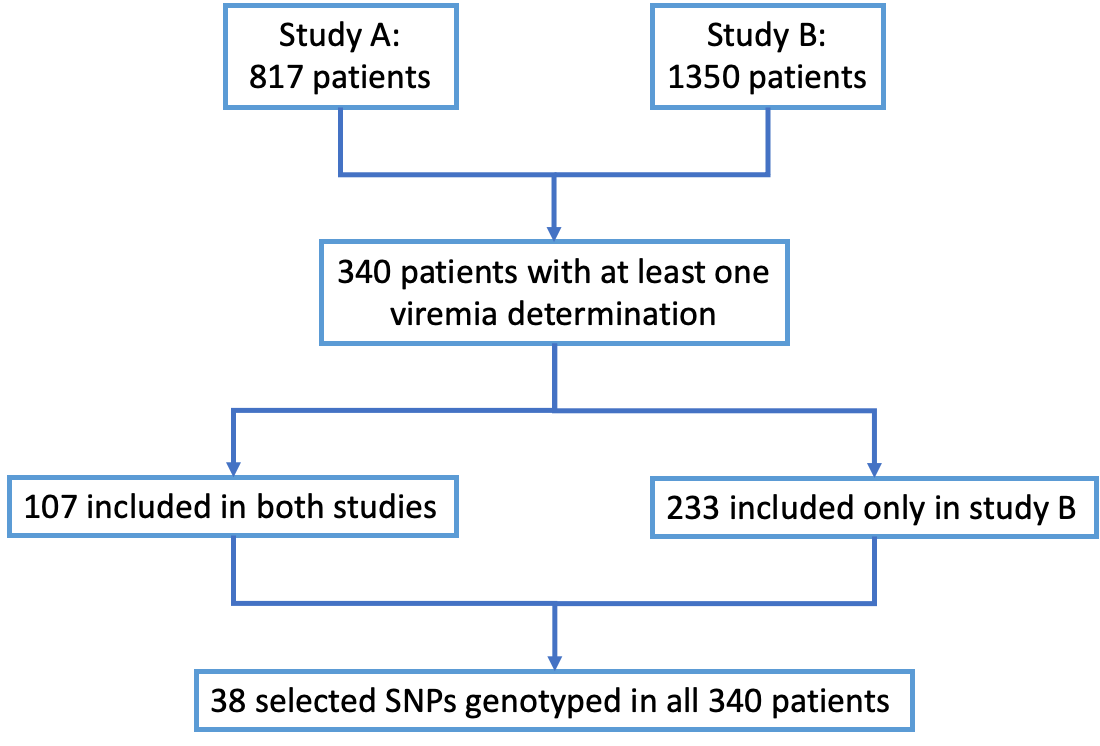


Supplementary Figure 1. Flow chart of patients included in the study
